# Supplementary material for: Gut microbial structural variation associates with immune checkpoint inhibitor response
Source: Nat Commun. 2023 Nov 16;14:7421. doi: 10.1038/s41467-023-42997-7 (PMC10654443; doi:10.1038/s41467-023-42997-7)
Supplement: Supplementary file 1 — Supplementary Information [file 41467_2023_42997_MOESM1_ESM.pdf]

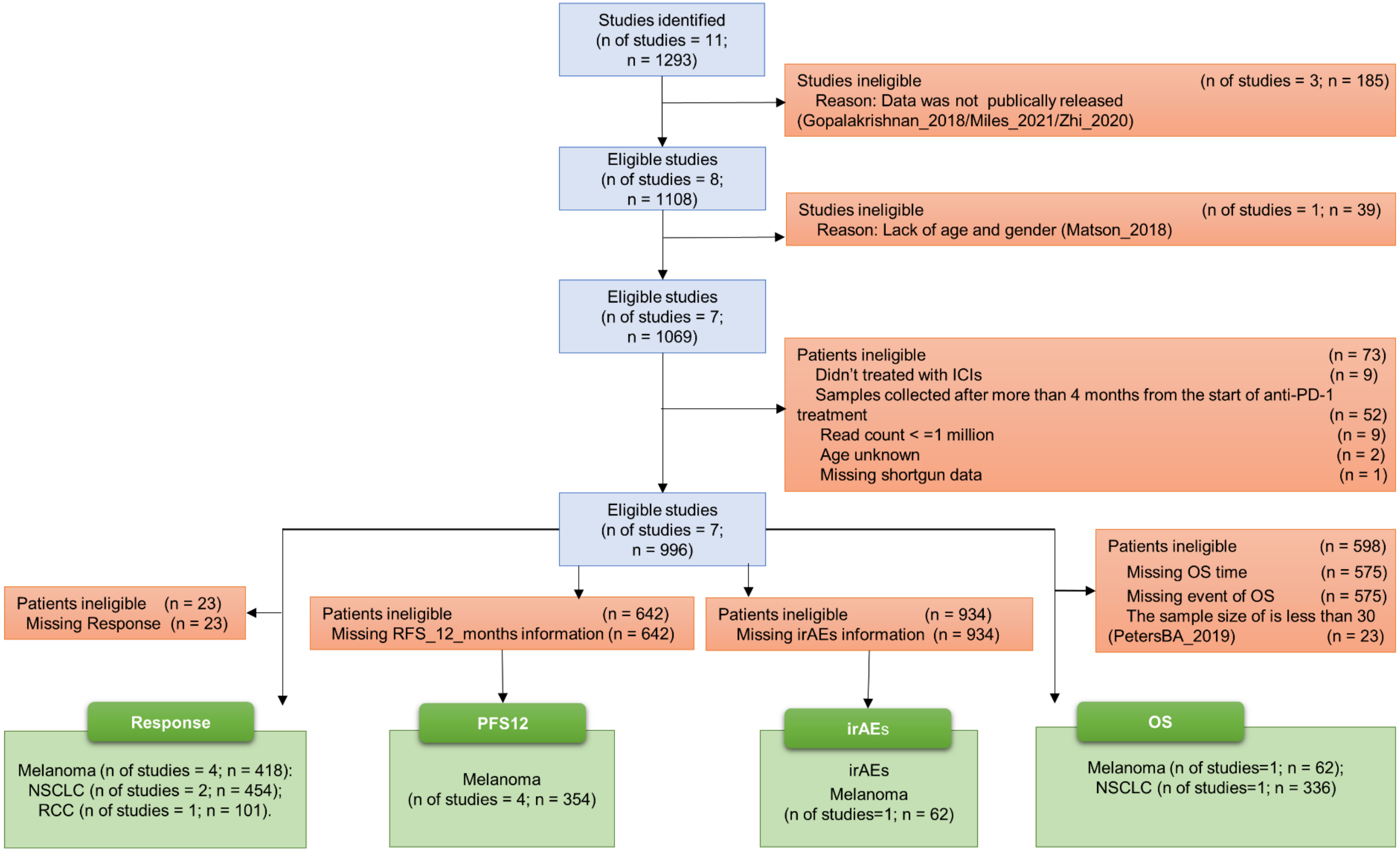
Studies identified (n of studies = 11; n = 1293)
Studies ineligible (n of studies = 3; n = 185)
Reason: Data was not publically released (Gopalakrishnan_2018/Miles_2021/Zhi_2020)
Eligible studies (n of studies = 8; n = 1108)
Studies ineligible (n of studies = 1; n = 39)
Reason: Lack of age and gender (Matson_2018)
Eligible studies (n of studies = 7; n = 1069)
Patients ineligible (n = 73)
Didn't treated with ICIs (n = 9)
Samples collected after more than 4 months from the start of anti-PD-1 treatment (n = 52)
Read count < =1 million (n = 9)
Age unknown (n = 2)
Missing shortgun data (n = 1)
Eligible studies (n of studies = 7; n = 996)
Patients ineligible (n = 23)
Missing Response (n = 23)
Patients ineligible (n = 642)
Missing RFS_12_months information (n = 642)
Patients ineligible (n = 934)
Missing irAEs information (n = 934)
Patients ineligible (n = 598)
Missing OS time (n = 575)
Missing event of OS (n = 575)
The sample size of is less than 30 (PetersBA_2019) (n = 23)
Response
Melanoma (n of studies = 4; n = 418): NSCLC (n of studies = 2; n = 454); RCC (n of studies = 1; n = 101).
PFS12
Melanoma (n of studies = 4; n = 354)
irAEs
irAEs Melanoma (n of studies=1; n = 62)
OS
Melanoma (n of studies=1; n = 62); NSCLC (n of studies=1; n = 336)

Fig. S1. A flowchart of the study that details the samples utilized at each stage of statistical analysis. OS: Overall survival. irAEs: immune-related adverse events. NSCLC: Non-small-cell lung cancer; RCC: Renal cell carcinoma. PFS12: progression-free survival at 12 months.

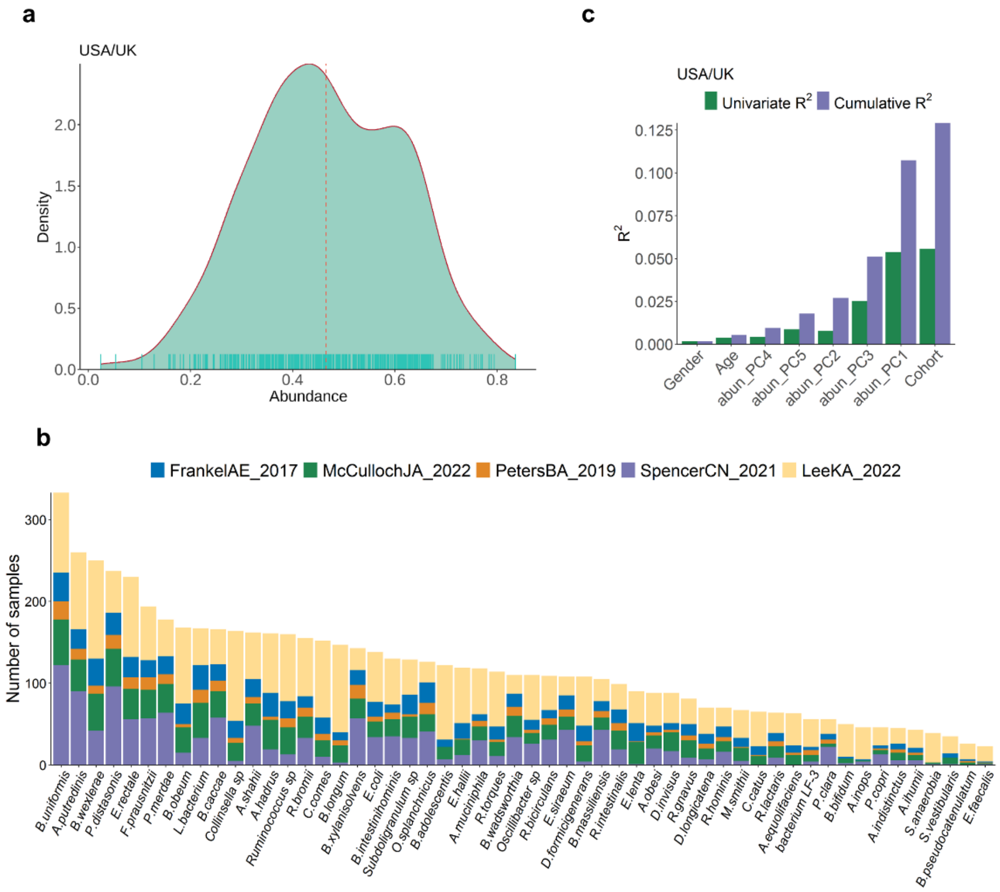

Fig. S2. Characterization of microbial SV profile in cohort from USA or UK. a) Distribution of the relative abundance of species detected with SVs. b) Numbers of samples with sufficient coverage across the reference genomes of each species. c) The proportion of SV-based genetic variance that is explained by basic phenotypic factors and microbial compositional factors.

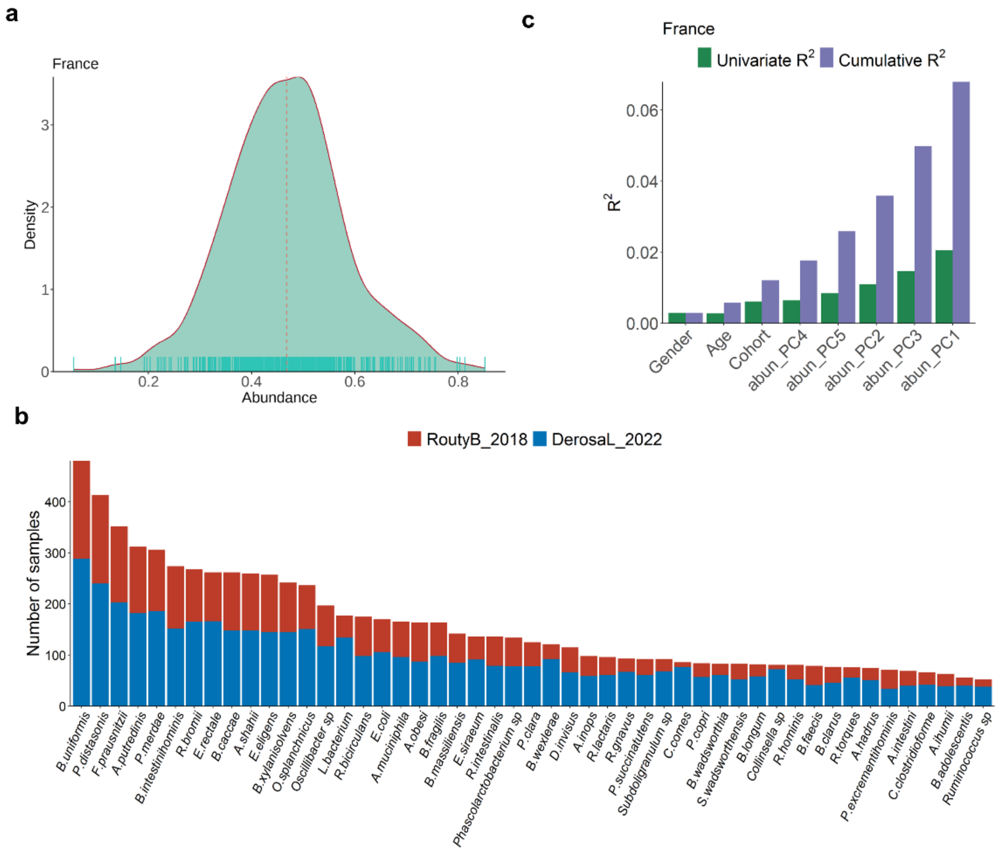

Fig. S3. Characterization of microbial SV profile in cohort from France. a) Distribution of the relative abundance of species detected with SVs. b) Numbers of samples with sufficient coverage across the reference genomes of each species. c) The proportion of SV-based genetic variance that is explained by basic phenotypic factors and microbial compositional factors.

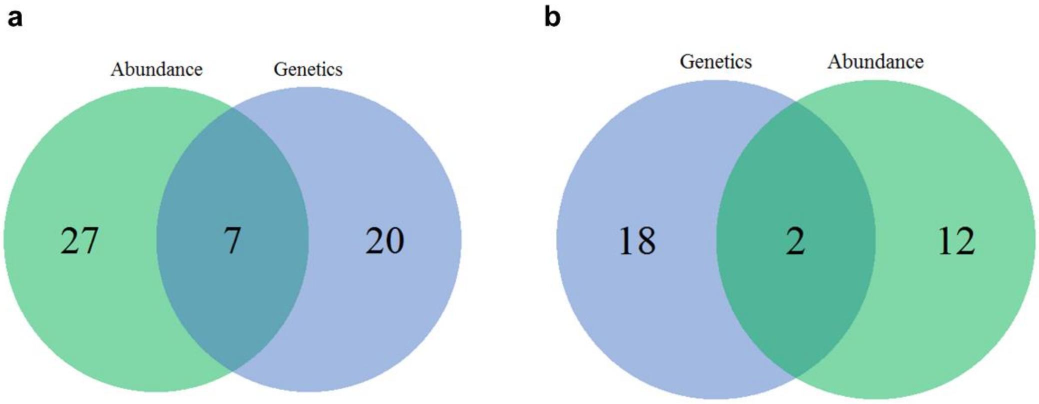

Figure S4. Number of significant associations with prognosis after ICI treatment for genetics and abundance in USA/UK cohorts (a) and France (b) cohort.

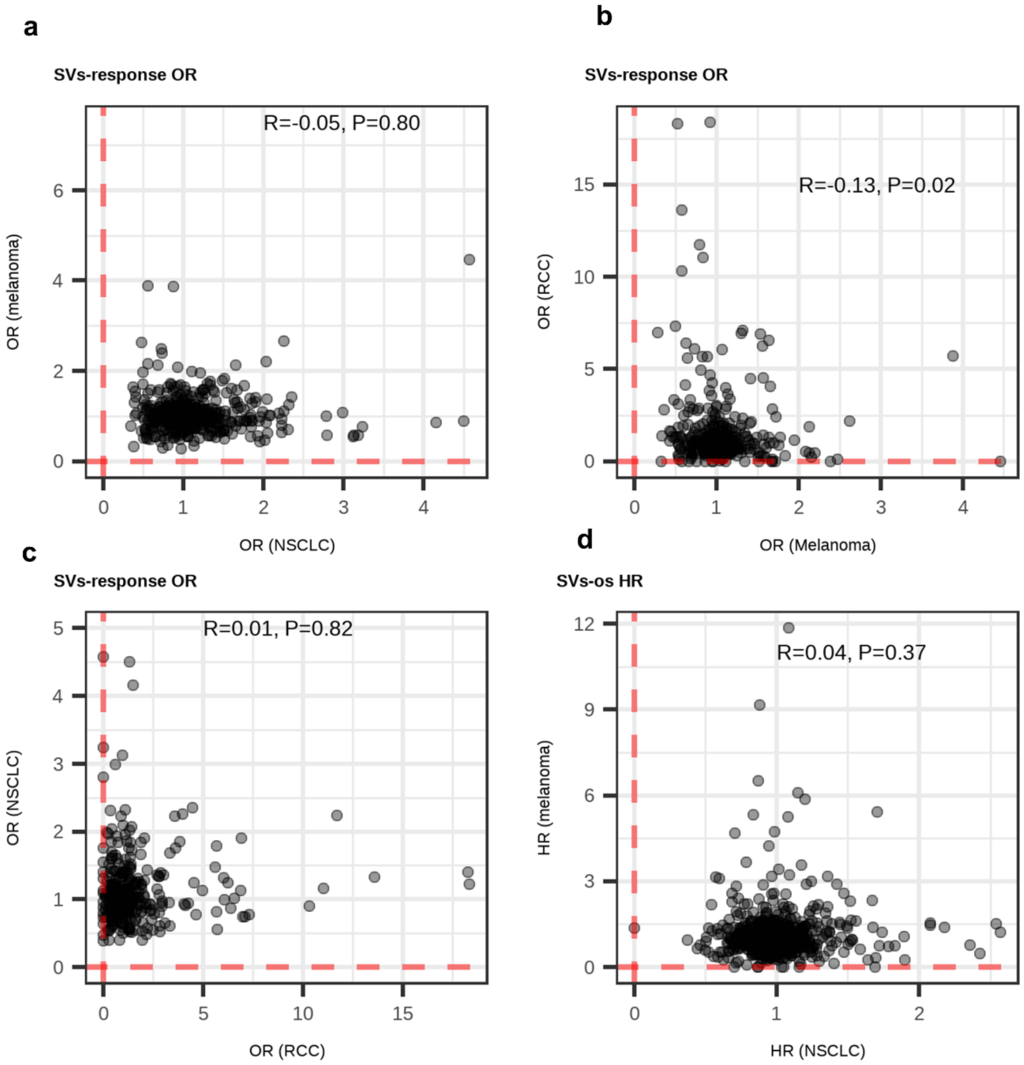

Fig. S5. The between-cancer type correlation of effect-sizes for associations with SVs (Spearman correlation).
