# Supplementary material for: Gut microbial structural variation associates with immune checkpoint inhibitor response
Source: Nat Commun. 2023 Nov 16;14:7421. doi: 10.1038/s41467-023-42997-7 (PMC10654443; doi:10.1038/s41467-023-42997-7)
Supplement: Supplementary file 3 — Description of Additional Supplementary Files [file 41467_2023_42997_MOESM3_ESM.docx]

**Description of Supplementary Data Files**

**Gut microbial structural variation associates with immune checkpoint inhibitor response**

Rong Liu^1,2,3,4*^, You Zou^5^, Wei-Quan Wang^1,2,3,4^, Jun-Hong Chen^1,2,3,4^, Lei Zhang^1,2,3,4^, Jia Feng^1,2,3,4^, Ji-Ye Yin^1,2,3,4^,Xiao-Yuan Mao^1,2,3,4^, Qing Li^1,2,3,4^, Zhi-Ying Luo^6,7^, Wei Zhang^1,2,3,4^*, Dao-Ming Wang^8,9^*

^+^co-corresponding author

Corresponding authors:

Rong Liu

E-mail: [liuronghyw@csu.edu.cn](mailto:liuronghyw@csu.edu.cn)

Wei Zhang:

E-mail: [csuzhangwei@csu.edu.cn](mailto:csuzhangwei@csu.edu.cn)

Dao-MingWang

E-mail: d.wang@umcg.nl

1. Department of Clinical Pharmacology, Xiangya Hospital, Central South University, 87 Xiangya Road, Changsha 410008, P. R. China;

2. Institute of Clinical Pharmacology, Central South University, Hunan Key Laboratory of Pharmacogenetics, 110 Xiangya Road, Changsha 410078, P. R. China;

3. Engineering Research Center of Applied Technology of Pharmacogenomics, Ministry of Education, 110 Xiangya Road, Changsha 410078, P. R. China;

4. National Clinical Research Center for Geriatric Disorders, 87 Xiangya Road, Changsha 410008, Hunan, P.R. China.

5. Information and Network center, Central South University, Changsha, 410083, P.R. China;

6. Department of Pharmacy, The Second Xiangya Hospital, Central South University, Changsha, PR China

7. Institute of Clinical Pharmacy, Central South University, Changsha, PR China

8. University of Groningen, University Medical Center Groningen, Department of Genetics, Groningen 9713AV, the Netherlands

9. University of Groningen, University Medical Center Groningen, Department of Pediatrics, Groningen 9713AV, the Netherlands

File name: Supplementary Data 1

Description: A) Summary of ICI metagenome studies exclude from this study; B) Distributions of clinical characteristics by study; C) assignment values of the clinical variables; D) The definition of candidate associations for different clinical outcomes per cancer types.

File name: Supplementary Data 2

Description: A) Information on the species detected with SVs of UK dataset (LeeKA_2022), related to Fig. 3a-2b. B) Information on the species detected with SVs of France dataset (DerosaL_2022), related to Fig. 3c-d.

File name: Supplementary Data 3

Description: A) Species-level associations of response with microbial genetic makeup (PERMANOVA) and relative abundance (logistic regression) for melanoma; B) Species-level associations of PF S>= 12 months with microbial genetic makeup (PERMANOVA) and relative abundance (logistic regression) for melanoma; C) Species-level associations of irAEs with microbial genetic makeup (PERMANOVA) and relative abundance (logistic regression) for melanoma. D) Species-level associations of response with microbial genetic makeup (PERMANOVA) and relative abundance (logistic regression) for NSCLC. E) Species-level associations of response to ICIs with microbial genetic makeup (PERMANOVA) and relative abundance (logistic regression) for RCC. Related to Fig. 4.

File name: Supplementary Data 4

Description: A) Associations (meta P < 0.05) between dSVs and response to ICIs of melanoma; B) Associations (meta P <0.05) between vSVs and response to ICIs of melanoma. C) Associations (meta P <0.05) between dSVs and pfs (≥12 months) of melanoma. D) Associations (meta P <0.05) between vSVs and pfs (≥12 months) of melanoma. E) Associations (meta P <0.05) between dSVs and immune-related adverse events of melanoma. F) Associations (meta P <0.05) between vSVs and immune-related adverse events of melanoma. G) Associations (meta P <0.05) between dSVs and overall survival of melanoma. H) Associations (meta P <0.05) between vSVs and overall survival of melanoma. I) Associations (meta P <0.05) between dSVs and response to ICIs of non-small cell lung cancer. J) Associations (meta P <0.05) between vSVs and response to ICIs of non-small cell lung cancer. K) Associations (meta P <0.05) between dSVs and overall survival of non-small cell lung cancer. L) Associations (meta P <0.05) between vSVs and overall survival of non-small cell lung cancer. M) Associations (meta P <0.05) between dSVs and response to ICIs of renal cell carcinoma. N) Associations (meta P <0.05) between vSVs and response to ICIs of renal cell carcinoma.
