# Supplementary material for: Gut microbial structural variation associates with immune checkpoint inhibitor response
Source: Nat Commun. 2023 Nov 16;14:7421. doi: 10.1038/s41467-023-42997-7 (PMC10654443; doi:10.1038/s41467-023-42997-7)
Supplement: Supplementary file 8 — Reporting Summary [file 41467_2023_42997_MOESM8_ESM.pdf]

nature portfolio

Corresponding author(s): Rong Liu

Last updated by author(s): 2023/10/18

Reporting Summary

Nature Portfolio wishes to improve the reproducibility of the work that we publish. This form provides structure for consistency and transparency in reporting. For further information on Nature Portfolio policies, see our Editorial Policies and the Editorial Policy Checklist.

Statistics

For all statistical analyses, confirm that the following items are present in the figure legend, table legend, main text, or Methods section.

n/a	Confirmed	
☐	☒	The exact sample size (n) for each experimental group/condition, given as a discrete number and unit of measurement
☐	☒	A statement on whether measurements were taken from distinct samples or whether the same sample was measured repeatedly
☐	☒	The statistical test(s) used AND whether they are one- or two-sided<br>*Only common tests should be described solely by name; describe more complex techniques in the Methods section.*
☐	☒	A description of all covariates tested
☐	☒	A description of any assumptions or corrections, such as tests of normality and adjustment for multiple comparisons
☐	☒	A full description of the statistical parameters including central tendency (e.g. means) or other basic estimates (e.g. regression coefficient) AND variation (e.g. standard deviation) or associated estimates of uncertainty (e.g. confidence intervals)
☐	☒	For null hypothesis testing, the test statistic (e.g. F, t, r) with confidence intervals, effect sizes, degrees of freedom and P value noted<br>*Give P values as exact values whenever suitable.*
☒	☐	For Bayesian analysis, information on the choice of priors and Markov chain Monte Carlo settings
☒	☐	For hierarchical and complex designs, identification of the appropriate level for tests and full reporting of outcomes
☒	☐	Estimates of effect sizes (e.g. Cohen's d, Pearson's r), indicating how they were calculated

Our web collection on statistics for biologists contains articles on many of the points above.

Software and code

Policy information about availability of computer code

Data collection	Raw metagenomics sequencing data of the seven datasets are publicly available from the European Genome-Phenome Archive via accession numbers (PRJNA397906, PRJNA541981, PRJNA762360, PRJEB22863, PRJNA770295, PRJNA751792, and PRJEB43119).<br>The raw metagenomic sequencing data underwent a data cleaning procedure to remove low-quality reads and host genome-contaminated reads with the usage of KneadData (version 0.6.1), Trimmomatic (version 0.39) and Bowtie2 (version 2.3.5.1). SVs were detected based on the high-quality metagenomic sequence reads with SGV-Finder algorithm. The taxonomic relative abundance of all samples utilized in this study was generated from high-quality metagenomic reads using Kraken2 (version 2.1.2) and Bracken (version 2.6.1). The reference genomes were also developed based on the Progenome Database. All statistical tests were performed with R (version 4.0.5).
Data analysis	The custom designed code used for statistical analysis is available via https://github.com/liuronghyw/ICIs_gut_microbe_SVs

For manuscripts utilizing custom algorithms or software that are central to the research but not yet described in published literature, software must be made available to editors and reviewers. We strongly encourage code deposition in a community repository (e.g. GitHub). See the Nature Portfolio guidelines for submitting code & software for further information.

Data

Policy information about availability of data

All manuscripts must include a data availability statement. This statement should provide the following information, where applicable:

- Accession codes, unique identifiers, or web links for publicly available datasets
- A description of any restrictions on data availability
- For clinical datasets or third party data, please ensure that the statement adheres to our policy

Raw metagenomics sequencing data of the seven datasets are publicly available from the European Genome-Phenome Archive via accession numbers (PRJNA397906, PRJNA541981, PRJNA762360, PRJEB22863, PRJNA770295, PRJNA751792, and PRJEB43119). All these studies have been previously approved by their respective institutional review boards.

Research involving human participants, their data, or biological material

Policy information about studies with human participants or human data. See also policy information about sex, gender (identity/presentation), and sexual orientation and race, ethnicity and racism.

Reporting on sex and gender	NA
Reporting on race, ethnicity, or other socially relevant groupings	NA
Population characteristics	NA
Recruitment	NA
Ethics oversight	NA

Note that full information on the approval of the study protocol must also be provided in the manuscript.

Field-specific reporting

Please select the one below that is the best fit for your research. If you are not sure, read the appropriate sections before making your selection.

☒ Life sciences ☐ Behavioural & social sciences ☐ Ecological, evolutionary & environmental sciences

For a reference copy of the document with all sections, see nature.com/documents/nr-reporting-summary-flat.pdf

Life sciences study design

All studies must disclose on these points even when the disclosure is negative.

Sample size	This bioinformatics analysis utilized publicly accessible metagenomics sequencing data from ICI trials. Raw gut metagenomic data of 996 ICI-treated patients from seven cohorts was gathered. We have collected almost all publicly available immune checkpoint inhibitor studies with metagenome data all over the world. As we listed in Table S1A, some datasets need requests, and we have failed to get permission to obtain the dataset after sending an email to the corresponding PIs.
Data exclusions	Samples didn’t receive ICI treatment or collected after more than 4 months of from the start of ICI treatment, or without matched clinical and metagenomics data available were filtered.
Replication	The associations between gut microbial SVs and hosts' clinical outcomes after ICI treatment were tested within different datasets. Then we focus on finding replicated associations between different cohorts.
Randomization	The randomization may conducted within the 7 publicly accessible ICI trials. Since we just re-analysis the clinical outcomes and metagenomic data of the above studies. It is not necessary for us to do randomization. When conduct association analysis, age and gender were included as covariates for adjustment.
Blinding	The blinding may conducted within the 7 publicly accessible ICI trials. Since we just re-analysis the clinical outcomes and metagenomic data of the above studies. It is not necessary for us to do blinding.

Reporting for specific materials, systems and methods

We require information from authors about some types of materials, experimental systems and methods used in many studies. Here, indicate whether each material, system or method listed is relevant to your study. If you are not sure if a list item applies to your research, read the appropriate section before selecting a response.

Materials & experimental systems

n/a	Involved in the study
☒	☐ Antibodies
☒	☐ Eukaryotic cell lines
☒	☐ Palaeontology and archaeology
☒	☐ Animals and other organisms
☒	☐ Clinical data
☒	☐ Dual use research of concern
☒	☐ Plants

Methods

n/a	Involved in the study
☒	☐ ChIP-seq
☒	☐ Flow cytometry
☒	☐ MRI-based neuroimaging
